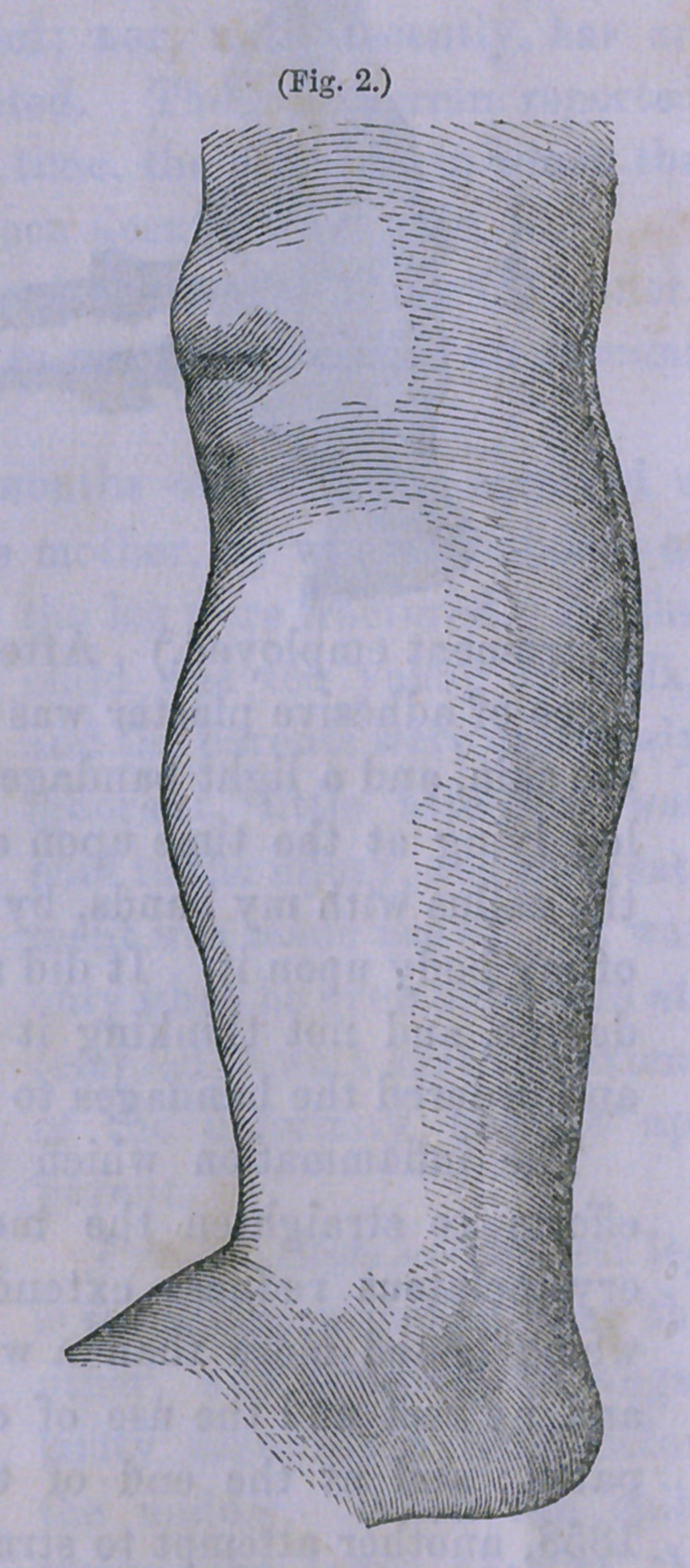# Report of a Case of Deformity from Fracture, Successfully Treated by a New Method*This Report was presented and read to the Society of Surgery, at one of its sittings, in October, 1858, by Baron Larrey, for the author. It was ordered t inserted entire in the bulletins, and was printed in the *Gazette des Hopitaux*.

**Published:** 1859-01

**Authors:** Daniel Brainard

**Affiliations:** Corresponding Member


THE CHICAGO

### MEDICAL JOURNAL.

VOL. II. JANUARY, 1859. No. 1.

### ORIGINAL COMMUNICATIONS.

ARTICLE I.

### REPORT OF A CASE OF DEFORMITY FROM FRACTURE, SUCCESSFULLY TREATED BY A NEW METHOD.*

(ADDRESSED TO THE SOCIETY OF SURGERY OF PARIS.)

BY DANIEL BRAINARD, M. D., CORRESPONDING MEMBER.

During the course of the year 1853, the author of this report published, at Paris, a *Memoire sur le traitement des fractures non reunies et des difformites des os*, in which was proposed a new method of treatment for certain of these deformities, such, for example, as irregularity of bones resulting from badly treated fractures. The method of treatment proposed consisted in weakening the bone by sub-cutaneous perforation, and causing it to soften by the inflammation thus excited, and then straighten-

* This Report was presented and read to the Society of Surgery, at one of its sittings, in October, 1858, by Baron Larrey, for the author. It was ordered t inserted entire in the bulletins, and was printed in the *Gazette des Hopitaux*.

ing it by pressure applied gradually or suddenly by the hands. The facts adduced in that *Memoire* in favor of this method of treatment were the results of experiments on the bones of living animals, principally dogs, and some figures were given of bones bent and partially fractured by such treatment.

I had not, however, at that time had occasion to apply the treatment on the human subject; nor, until recently, has an opportunity for so doing presented. The case herein reported is the first and, at the present time, the only one in which the application of the method has been deemed advisable.

CASE.—May 15th, 1858, a stout boy, named John O'Connor, aged three years, was brought to me for treatment on account of a deformity of the left leg.

*History.*—When but three months old, this boy received a severe fall from the arms of his mother, by which the bones of the leg were fractured. As the child was too young to walk, and the parents were extremely ignorant, little attention was paid to the injury, and no treatment was made use of. It was only when he grew older and attempted to walk that the extent of the deformity became apparent.

(Fig. 1.)

*Present State.*—The left leg is three inches shorter than the other, and presents an angularity forwards a little below the middle. When the child walks, the lower part of the tibia rests upon the dorsum of the foot. There is no swelling or tenderness at the point of fracture. (The appearance of the member at this time is perfectly represented by the photographic figure marked 1.)

*Operation.*—The child having been placed under the influence of chloroform, a perforator, one-fourth of an inch in breadth, was passed in two different directions through the tibia at the point of fracture, but a single puncture being made through the skin. (Fig. 3 represents the form and is half the size of the

(Fig. 3.)

instrument employed.) After the perforator was withdrawn, a piece of adhesive plaster was placed upon the puncture through the skin, and a light bandage placed around the member. The leg lying at the time upon a firm bed, I attempted to rupture the callus with my hands, by throwing nearly the whole weight of my body upon it. It did not, however, yield in the slightest degree, and not thinking it safe to use more force I desisted, and ordered the bandages to be kept wet with cold water.

The inflammation which followed this operation, and the efforts to straighten the member, was considerable, and an erysipelatous redness extended from the ankle to the knee, which lasted more than a week. There was no suppuration, and by rest and the use of evaporating lotions, this was dissipated; and at the end of ten days, viz: on the 25th May, 1858, another attempt to straighten the leg was made.

Although hoping for a favorable change, I was somewhat surprised to find that a very moderate degree of force, applied by the hands, was sufficient to cause the callus to give. A carved wooden splint, well padded, with a foot piece, was now placed behind the leg, and secured to it by a roller drawn across the angular projection as tightly as could be borne. This giving rise to no pain, the bandage was re-applied, every three days at first, afterwards once a week, for four weeks, at the end of which time the leg was quite straight, except a slight overlapping of the fragments. During this part of the treatment, the boy walked about with the splint on his leg and suffered no pain. The parents were directed to press upon the projection daily with the hands.

(Fig. 2.)

At the present time, August 25th, more than three months after the operation, the leg presents the appearance represented in the photographic figure marked 2. The splints have been discontinued; there is no swelling nor tenderness of the part; the boy walks well, and the cure seems complete.

The means hitherto resorted to for the cure of deformities, such as that presented in the above case, are section or resection of the callus, and extension, sudden or gradual. It is not my intention to offer any reflections upon the value of these methods; but it may safely be asserted that they are all severe, and attended with danger, excepting the last, which is insufficient in all except recent cases.

The method which I have proposed, and of the successful application of which the above case is an example, is founded upon the fact, well known at present, that bone is softened by inflammation. In the *Memoire* to which I have referred, I have attempted to show that by perforation and the inflammation thence resulting, bones may be so much weakened as to be bent and partially broken like stems of green wood with the application of but little force. This case shows that the same is true in a more marked degree of callus.

Surgeons have long felt the want of a means to soften deformed callus. The use of fomentations, baths, frictions, applications of oil, etc., are sufficient proofs of this fact.

M. Malgaigne, in his classical work on fractures (page 333), says, an important question to be solved is to know if our art possesses any means for softening callus, so as to cause it to yield more easily; and after referring to the means above enumerated, and others, he concludes that none of them are deserving of confidence. It is this want which I hope to supply by the introduction of a new method of treatment.

In conclusion, permit me to add that I do not advise the resort to this method in every case. The age and good health of this patient, the superficial situation of the tibia (the only bone operated on), are all circumstances favorable to success; still, I am not without the hope that it will be found applicable in a considerable variety of cases hitherto regarded as incurable, or in which more severe operations are regarded as necessary.

CHICAGO, August, 1858.

### REMARKS ON THE MEANS HERETOFORE EMPLOYED FOR REMEDYING THE DEFORMITY RESULTING FROM FRACTURE.

It may interest many of the readers of the *Journal* to present, in a concise form, a view of the principal means hitherto invented for the remedy of these deformities. They are—

I. MECHANICAL FORCE GRADUALLY APPLIED.

This may be done by simple extension, when union has taken place with the bones, in a straight direction. Although this has been recommended, and is still enumerated among the means to be tried, it is evident that it can only be applicable where the union is still soft. Lateral compression may be resorted to in angular deformities with better chances of succeeding, since the fragments can, to a certain degree, be employed as levers. This method is applicable for four or six weeks after the union has seemed firm, and in some cases longer still. Various mechanical contrivances have been resorted to in these cases, but they have not been found generally useful.

II. Section or Resection of the Callus.

This consists in cutting down upon the callus and dividing it with a saw or chisel, or in sawing out a wedge-shaped piece, as has been recommended by Barton in certain forms of anchylosis. In many cases of firmly consolidated fractures this is the only recourse except amputation. Were I to meet with a case in which the callus could not be divided with the instrument I have described by the sub-cutaneous method (and I think there may be many where only a smaller one could be made to perforate perfectly formed callus), I should have the shaft of it made sufficiently strong to be used as a chisel, and after placing the point of it in contact with the callus, strike it with a mallet until the perforation is accomplished. Resection or section, unless by the sub-cutaneous method, is a severe operation—converts a simple or united into a compound fracture. The dangers of such a proceeding it is desirable to avoid.

III. Forcible Rupture.

Where a bone has united in an unfavorable position, and this is discovered before the callus has become too firm to be broken by the hands of the surgeon, the best way of proceeding is to put the patient under the influence of chloroform, rupture the callus, re-adjust and dress the fracture. I have succeeded perfectly by this method in two cases brought to me in a bad condition, and have met with another where the patient made a false step in walking, fell and re-fractured the leg. A re-adjustment remedied the deformity which existed previously to the second fracture.

IV. The Seton.

Weinhold, in a case of deformity after fracture of the femur, drilled a hole through the callus and inserted a seton. About the seventh week the callus began to yield, and the limb was made good by extension.* The use of the seton for this purpose is founded upon physiological principles perfectly correct. My experiments have proved that foreign bodies of every kind left in contact with bones cause absorption, particularly if the body be not metallic, and causes suppuration. It is well known that

* Malgaigne's Operative Surgery, p. 188.

fragments of bone in comminuted fracture prevent union from taking place. The justice of Dr. Weinhold's views in this particular is the more remarkable when contrasted with those of so many eminent surgeons, who have recommended not only setons, but pegs of ivory, etc., to be placed and kept between the fragments of fractured bone for the purpose of obtaining union.

## Figures and Tables

**Fig. 1. f1:**
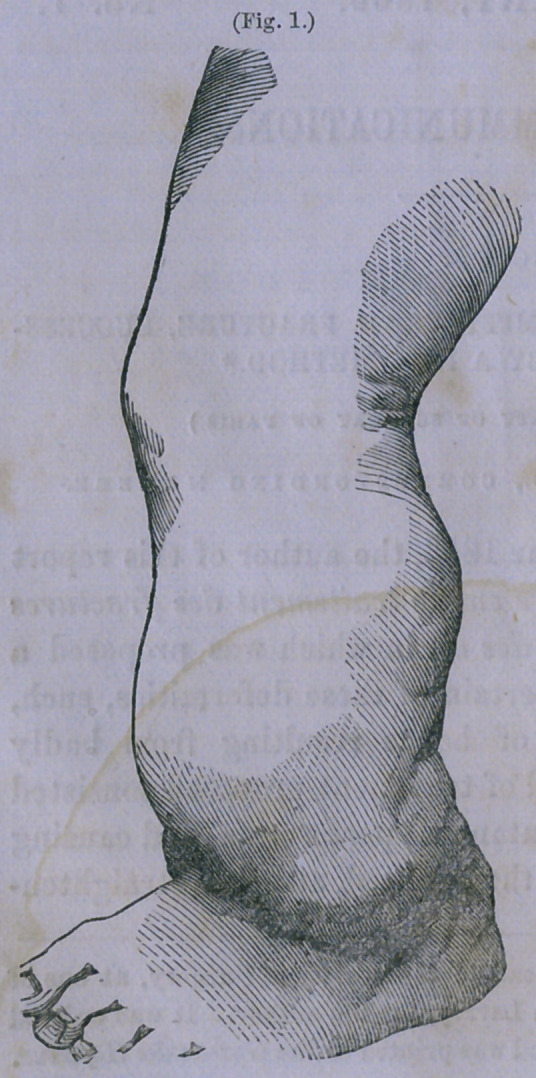


**Fig. 3. f2:**
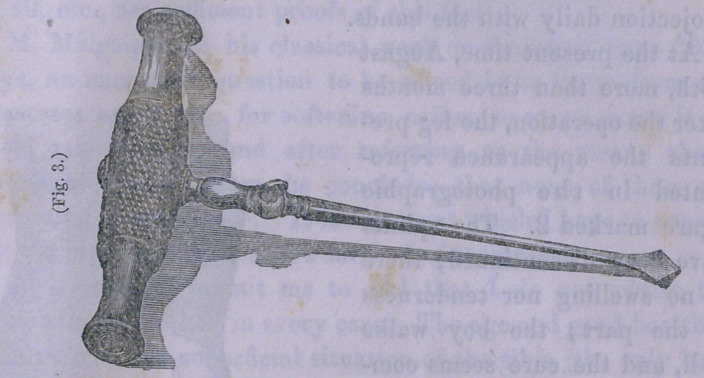


**Fig. 2. f3:**